# Silymarin, a Popular Dietary Supplement Shows Anti–*Candida* Activity

**DOI:** 10.3390/antibiotics8040206

**Published:** 2019-10-31

**Authors:** Monika Janeczko, Elżbieta Kochanowicz

**Affiliations:** Department of Molecular Biology, The John Paul II Catholic University of Lublin, ul. Konstantynów 1i, 20-708 Lublin, Poland; mazure@kul.pl

**Keywords:** antifungal activity, *Candida*, diet supplementation, *Silybum marianum*, silymarin

## Abstract

Silymarin is a complex of plant-derived compounds obtained from the seed shells of the milk thistle (*Silybum marianum)*. It is used in medicine primarily to protect the liver. The mixture contains mainly flavonolignans, with silybin as a paramount bioactive component of the extract. This article presents the potential health benefits for silymarin as an antifungal drug against five references strains: *C. albicans*, *C. glabrata*, *C. parapsilosis, C. tropicalis*, and *C. krusei* with MIC (minimum inhibitory concentration) values ranging from 30 to 300 µg/mL. Additionally, this study revealed that the compound suppressed the growth of cells of most of the tested clinical *Candida albicans* strains with MIC values between 30 and 1200 µg/mL. Based on the fractional inhibitory concentration index (FICI), the combination of silymarin with antifungal drugs caspofungin, fluconazole, and amphotericin B did not significantly change the MIC values for the tested *Candida* strains. Furthermore, no antagonistic reactions were observed in any combination of drugs. In addition, this substance shows anti-virulence properties including the destabilization of mature biofilm and the inhibition of the secretion of hydrolases. qRT-PCR-based experiments demonstrated that the *SAP4* gene involved in virulence was downregulated by silymarin. These results indicate completely new advantages of dietary supplementation with this natural plant extract.

## 1. Introduction

Nowadays, dietary supplements are very popular and commonly used throughout the world. Their goal is to supplement the normal diet with vitamins, minerals, or other nutrients (single or complex) that have a nutritional or other physiological effect. A number of them are obtained from plants [1].

Milk thistle (*Silybum marianum*, family: *Asteraceae*) is a medical plant that has been used for at least 2000 years. In ancient Greece, India, and China, *Silybum marianum* was administered to cure liver and gallbladder diseases, in addition to the detoxification of organisms. The bioactive extract of milk thistle seeds and fruits, silymarin was classified by the World Health Organization as an official drug with hepatoprotective properties in the 1970s. Currently, silymarin is also commonly used as a dietary supplement, particularly in supporting liver function, but also as a medicine employed in many liver diseases and metabolic syndromes including obesity, diabetes, hypertension, and dyslipidemia. *Silybum marianum* is found mostly in Southern Europe and Asia [2]. 

Natural silymarin is a unique mixture containing flavonolignans, especially silybin, isosilybin, silychristin, isosilychristin, silydianin, and silimonin. In addition to flavonolignans, it also includes other flavonoids (quercetin, dihydrokaempferol, kaempferol, apigenin, naringin, eriodyctiol, and chrysoeriol) as well as 5,7-dihydroxy-chromone, dehydroconiferyl alcohol, solid oils, tocopherol, sterols, sugars, and proteins. However, the highest concentration in the extract has been shown by silybin (about 50–70%), which is also the main bioactive component of natural sylimarin. In conventional and standardized pharmaceuticals, silymarin is a complex of four flavonolignans, namely silybin, isosilybin, silydianin, and silychristin. The concentration of silybin in these products is typically in the range of 20–40% [3].

The hepatoprotective effect of silymarin results from its numerous biological activities (e.g., antioxidative, antifibrotic, anti-lipid peroxidative, anti-inflammatory, and immunomodulatory activities) as well as involvement in liver regeneration mechanisms. Supplementation of the diet with silymarin preparations are recommended to subjects exposed to liver cirrhosis and steatosis, inflammation of the organ, intensive antibiotic and drug treatment, digestive system disorders, toxins, and to be used by elderly people [2].

In recent years, many studies have demonstrated that, besides hepatoprotection, flavonolignans possess various healthy properties. New derivatives or new drug combinations of silymarin may result in the achievement of antiviral, antimalarial, anticancer, antidiabetic, neuroprotective, and neurotropic activities [4]. This article presents the potential health benefits of anti-*Candida* silymarin activity. 

*Candida* species are the most frequent source of fungal infection worldwide. *Candida albicans, Candida glabrata, Candida parapsilosis, Candida krusei*, and *Candida tropicalis* are major fungal pathogens responsible for both systemic and mucosal infections in humans. Opportunistic fungal infections affect mainly immunocompromised patients such as transplant recipients, cancer patients, and HIV/AIDS patients. *Candida* spp. displays a number of virulence properties that contribute consequentially to pathogenicity including the ability to adhere to host cells, secretion of degradative enzymes (e.g., phospholipases, aspartyl proteases), adaptation to the pH level, biofilm formation, immune system avoidance and phenotype switching, and morphological transition from yeast to pseudohyphal and hyphal filaments [5]. The limited treatment options in fungal diseases caused by resistant microorganisms have created the need to search for new ways for effective therapy. Enormous therapeutic potential is exhibited by many herbal extracts [6].

## 2. Results and Discussion

In order to verify the antifungal activity of silymarin, five of the most frequent *Candida* species were used for the tests. The broth microdilution assay revealed that silymarin inhibited the growth of all tested reference *Candida* strains. The minimal inhibitory concentration values were in the range of 30–300 µg/mL (Table 1). The strongest effect of silymarin was demonstrated for *C. albicans* and *C. krusei*, while its activity against *C. glabrata* and *C. tropicalis* was ten times less potent. The MICs of amphotericin B against the reference strains of *Candida* were 3.12–12.5 µg/mL. Susceptibility of yeast strains to caspofungin ranged from 0.09 to 0.78 µg/mL. Fluconazole completely inhibited the growth of *C. albicans* and *C. parapsilosis* at a concentration of 0.39 µg/mL and *C. tropicalis* at the concentration of 3.12 µg/mL 

**Figure 1:**
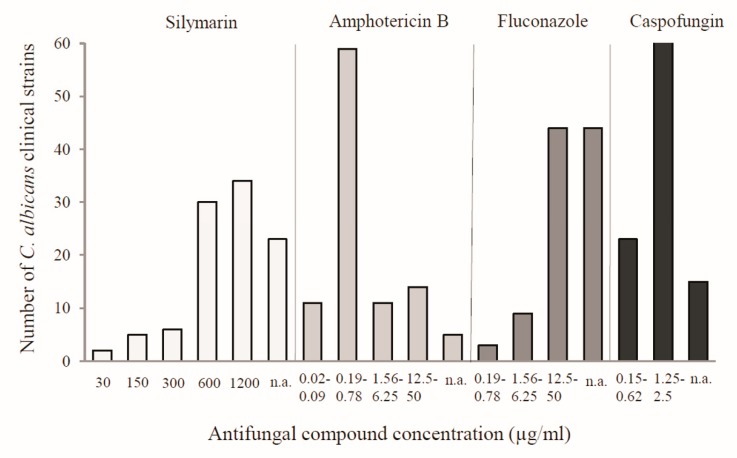
Influence of silymarin and antibiotics on the growth of clinical isolates of *C. albicans* expressed as MIC (minimal inhibitory concentration). n.a.—no activity at the concentration range used.

Furthermore, the effectiveness of silymarin against the clinical isolates of *C. albicans* was confirmed. The study was performed on 100 strains, which were isolated from the vaginas of gynecological patients. Silymarin showed efficacy against 77 tested clinical isolates with MIC in the range of 30–1200 µg/mL, while 23 isolates were not affected in the tested range of concentrations. Complete inhibition of cell growth for most strains occurred at the high concentrations of silymarin, namely at 600 µg/mL (30 isolates) and 1200 µg/mL (34 isolates). The MIC values for the reference antibiotics (i.e., amphotericin B, fluconazole, and caspofungin) ranged from 0.02 to 50 µg/mL, from 0.19 to 50 µg/mL, and from 0.15 to 2.5 µg/mL, respectively (Figure 1). The highest number of strains was inhibited by amphotericin in the concentration range of 0.19–0.78 µg/mL and by caspofungin in the concentration range of 1.25–2.5 µg/mL. Fluconazole was the most effective at concentrations from 12.5 to 50 µg/mL. Among the tested compounds, the highest percentage of resistant strains was found for fluconazole at the concentration range used.

As demonstrated in previous studies on natural compounds, phenols such as flavonoids and lignans have shown their antimicrobial potential [7]. Silymarin, which is composed mainly of flavonolignans, has also been reported to show antimicrobial activities against various microorganisms [8,9,10,11,12,13,14]. Recently, the antifungal effects of silymarin against clinical strains of *C. albicans*, *C. krusei*, and *C. tropicalis* have been demonstrated. The data reported in the literature by Rakelly de Oliveira showed that clinical strains of *Candida* were sensitive to its effects as the MIC values were 1024 µg/mL for all of the tested isolates [11]. Similarly, the MIC value for the most sensitive clinical strains was 1200 µg/mL in the present study (Figure 1).

The possibility of invading host tissues, survival, and existence in the host organism, and development of *Candida* spp. infections are facilitated by virulence factors [15]. In this study, silymarin was confirmed as an active substance against *Candida* adhesion, the formation of biofilms, and the secretion of hydrolytic enzymes (i.e., phospholipases and proteases). 

Cell adhesion is the first and critical stage of fungal infection and biofilm formation. *Candida* cells are capable of adherence to other *Candida* cells, other microorganisms, host cells, and abiotic surfaces. It is a complex, multifactorial process involving several proteins (adhesins) secreted by fungal cells. The ability to adhere to host tissues has been described in all species of the *Candida* genus, but in comparison with *C. albicans*, *C. tropicalis*, and *C. parapsilosis*, lower adhesive properties have been detected in *C. glabrata* [6,15]. In the recent years, many efforts have been made to explore new and effective natural compounds with anti-adhesion and anti-biofilm development effects [7,16,17,18]. Tests carried out to determine the anti-adhesive properties of silymarin have proven that the compound did not inhibit the adhesion of *Candida* cells. 

**Figure 2:**
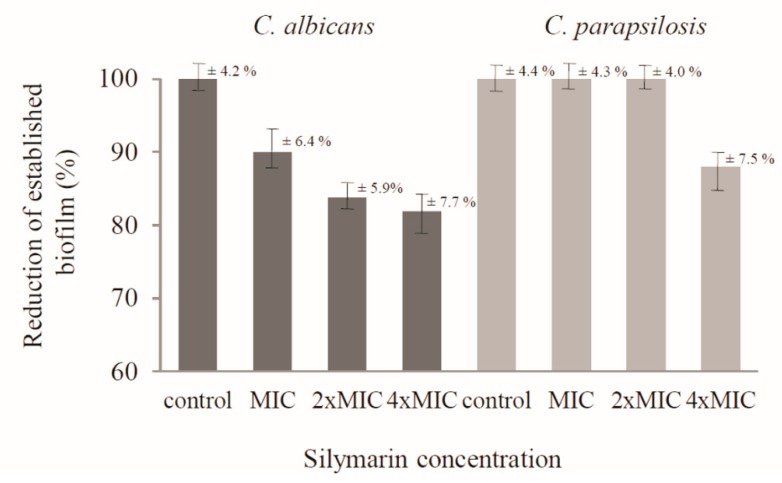
Influence of silymarin on established biofilms of *C. albicans* and *C. parapsilosis*.

In turn, this study revealed that silymarin destabilized mature biofilms produced by *C. albicans* and *C. parapsilosis* cells. Silymarin caused a reduction of formed *C. albicans* biofilm at the concentration corresponding to the MIC (30 µg/mL) to 90 ± 6.4%, while at higher concentrations, namely 2× MIC and 4× MIC, the reduction of biofilm biomass even reached 83.8 ± 5.9% and 81.9 ± 7.7%, respectively. At the three tested concentrations (MIC, 2× MIC, and 4× MIC), silymarin destabilized the biofilm formed by *C. parapsilosis* cells only at the highest concentration (240 µg/mL) to 88 ± 7.5%. In turn, no activity was demonstrated against biofilms formed by the other *Candida* reference strains. The results of biofilm quantification using crystal violet staining are presented in Figure 2.

Biofilm is a biological consortium with an extraordinary degree of organization, in which yeast cells form structured, coordinated, and functional communities settled in a self-secreted extracellular matrix. The National Institute of Health in the United States estimates that approximately 80% of infections are associated with biofilm etiology including *C. albicans* infections [19]. Furthermore, many chronic diseases such as otitis media, periodontitis prostatis, and native valve endocarditis seem to be caused by microorganisms associated with biofilm [20]. In comparison to planktonic cells, mature biofilm is much more resistant to antimicrobial agents and host immune system. Identification of new antifungal agents including those acting on biofilm cells seems to be crucial in the effective fight against *Candida* infections. Aside from commercial antibiotics, especially from the echinocandin class, anti-biofilm effects are shown by natural phythochemicals. Such properties are exhibited by eugenol, thymol, menthol, baicalin, berberine, farnesol, and cinnamaldehyde [21]. This study has confirmed that silymarin is another natural plant product with such desirable activity.

Hydrolytic enzymes are important virulence factors of *Candida* species. *C. albicans* expresses three different classes of secreted hydrolases: phospholipases, proteases, and lipases [15]. The effect of silymarin on the hydrolytic activity of *C. albicans* was detected for phospholipases and proteinases. 

**Figure 3:**
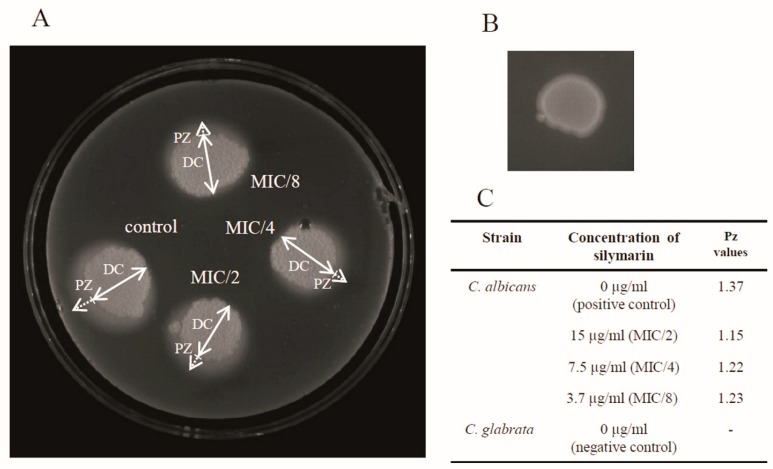
Egg yolk assay for phospholipase activity. (**A**) *C. albicans* unexposed to silymarin (as a positive control) or exposed to 15 µg/mL of silymarin. DC: diameter of the yeast colony; PZ: precipitation zone. (**B**) *C. glabrata* (as a negative control); (**C**) Pz values depending on the concentration of silymarin.

Phospholipases degrade phospholipid constituents of the host cell membrane, leading to disruption of the host cell. In addition, they modify surface features that facilitate adherence and subsequent infection [22]. *C. albicans* secrete phospholipases from four different classes (A–D), but only five members of class B (*PLB1-5*) are extracellular and contribute to pathogenicity [23]. As reported by Price and coworkers, 55% of *C. albicans* isolates from blood, 50% from wound infections, and 30% from the urine of subjects with serious *Candida* infections produced phospholipase [24]. The phospholipase activity of *C. albicans* is shown in Figure 3A. The Pz values of *C. albicans* exposed to silymarin at three different sub-therapeutic concentrations ranged from 1.15 to 1.23, and were similar to that of the positive control of *C. albicans* (Pz value was 1.63) (Figure 3C). The treatment with the 15 µg/mL, 7.5 µg/mL, and 3.7 µg/mL dilutions of silymarin reduced the extracellular phospholipase to 83.9%, 89%, and 89.8%, respectively. The differences in the Pz values between these concentrations were not significant. The negative control isolate of *C. glabrata* was negative for phospholipase (Figure 3B).

**Figure 4:**
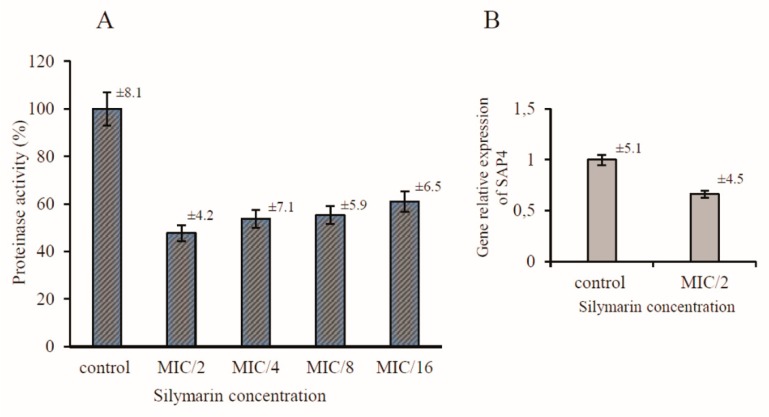
Effect of silymarin on proteinase activity (**A**) and on the relative expression of *SAP4* by qRT-PCR (**B**). Level of gene expression displayed after normalization with the internal control housekeeping gene *ACT1*.

*C. albicans* secrete aspartic proteinases (Saps) comprising of ten members, Sap 1–10. Proteinases Sap 1–8 are secreted and released to the extracellular space, whereas Sap9 and Sap10 are bound to the cell surface [25]. The activity of Saps is regarded as a virulence factor due to the observed expansion of Sap-encoding genes compared with less pathogenic relatives. Moreover, the proteolytic activity of other non-pathogenic *Candida* species is generally lower, which suggests that virulence is correlated with the level of Sap production [26]. Therefore, Saps are potential targets for the development of novel anti-*C. albicans* drugs. The casein assay was used to determine the effect of silymarin on the secretion and activity of proteinases from *C. albicans*. The decrease in the Sap activity after the treatment with the tested compound was shown as a percentage of the dimethyl sulfoxide (DMSO) control. As shown in Figure 4A, silymarin reduced the Sap activity to approximately 47.7 ± 4.2%, 53.8 ± 7.1%, 55.3 ± 5,9%, and 61 ± 6.5% in the MIC/2, MIC/4, MIC/8, and MIC/16, respectively, when compared with the control cells. The reduced activity of proteinases proved that silymarin prevented the secretion of these enzymes by the *C. albicans* cells. In the present work, we sought to further investigate the molecular mechanism supporting the silymarin-induced inhibition of *C. albicans* Saps. 

The pathogenesis of various forms of candidiasis depends on the differential and temporal regulation of the expression of genes associated with dimorphism, adhesion, and secretion of enzymes. To explore the potential mechanism of the inhibition of Sap secretion by silymarin, the expression of the *SAP4* gene was measured by real-time PCR. A more recently published study demonstrated that the *SAP4* gene is almost exclusively expressed during hyphal formation at neutral pH and at 30–37 °C [27,28]. The expression of *SAP4* in *C. albicans* cells was inhibited in the presence of silymarin during growth in conditions conducive to virulence. The relative expression of this gene was reduced to 0.663 when compared to the control silymarin-untreated cells (Figure 4B). The transcription inhibition of one of the genes encoding aspartyl proteinases seems to have a direct effect on the reduction of the secretion of Saps. Many reports indicate that natural herbal products contribute to the inhibition of the expression of critical adhesion and hyphal growth-associated genes [29,30,31].

With the use of the checkerboard method, silymarin was tested for possible antibiotic-modifying activity in combination with the antifungal drugs amphotericin B, caspofungin, and fluconazole. The interaction between the combined drugs was determined using the fractional inhibitory concentration index (FICI). This index was defined as FICI ≤ 0.5 (synergy), 0.5 < FICI ≤ 4 (no interaction), or FICI > 4 (antagonism). For four strains, *C. albicans*, *C. parapsilosis, C. glabrata*, and *C. tropicalis*, the FICI values of the silymarin/antibiotic combination varied primarily from 0.74 to 2. Therefore, these results showed indifferent interactions. The amphotericin B/silymarin and caspofungin/silymarin combinations showed a synergistic effect only in the case of *C. krusei*, with the FICI values of 0.35 and 0.39, respectively. Then, no antagonism was observed again. Due to the resistance of *C. glabrata* and *C. krusei* to fluconazole, the combination testing this antibiotic with silymarin was abandoned. The FICI values of the antifungal agents tested in combination are summarized in Table 2. 

To counteract the difficulties or failure of the conventional therapy of infections caused by drug-resistant organisms, innovative therapeutic strategies are often required. An innovative solution that increases the effectiveness of the treatment of microbial infections is combination therapy including a simultaneous use of several drugs and antimicrobials. It has been proven that the use of commercial antibiotics with herbal medicinal products is often an effective alternative in the treatment of diseases caused by bacteria, viruses, and fungi. In addition, it is an economically viable option as it eliminates the expensive and time-consuming process of developing new drugs. Drug interoperability (synergism) gives beneficial therapeutic effects (i.e., increased drug efficacy as well as reduced therapeutic dose and thus its toxicity) while increasing or maintaining the same efficacy and minimizing or slowing drug resistance [32].

At present, the anti-candidiasis drugs are limited to a few classes including azoles, polyenes, echinocandins, and allylamines. The increased multidrug resistance necessitates novel strategies for new effective antifungal therapy. Combinations of antifungal drugs and anti-virulence phytocompounds have proven to be an effective strategy to reduce drug resistant and repurpose known antifungals. Synergistic drug combinations provide a new option for antifungal drug discovery and more potent control against fungal infections. This mode of action against *C. albicans* is exhibited, for instance, by thymol and carvacrol in combination with fluconazole; berberine with fluconazole, miconazole, or amphotericin B; farnesol with fluconazole, ketoconazole, miconazole, or amphotericin B; eugenol with amphotericin B or fluconazole; honokiol with fluconazole; glabridin with ketoconazole; baicalein with amphotericin B; and terpenes with fluconazole [32]. As demonstrated in previous studies, silymarin exerted an antagonistic effect in combination with nystatin against *C. albicans*, *C. tropicalis*, and *C. krusei* and no significant effect in combination with mebedazole [11].

## 3. Materials and Methods

### 3.1. Candida Strains, Media, and Growth Conditions

In this study, five reference strains of *Candida*: *C. albicans* (ATCC 10231), *C. parapsilosis* (ATCC 22099), *C. glabrata* (ATCC 15126), *C. krusei* (ATCC 14243), and *C. tropicalis* (ATCC 13803), and 100 clinical strains of *Candida albicans* from gynecological patients were used. The hospital strains were identified using VITEK 2 YST IC CARDS (Biomerieux). For culture, the strains were grown in Sabouraud dextrose broth (Biocorp, Poland) at 30 °C with agitation (200 rpm) or on Sabouraud dextrose agar medium (Biocorp, Poland) at 30 °C. RPMI-1640 medium (with 1-glutamine and phenol red, without bicarbonate) (Sigma-Aldrich, USA) was buffered with 0.165 M 3-(N-morpholino) propane sulfonic acid (MOPS) (Sigma-Aldrich, USA) to pH 7.0. Stock solutions of silymarin (Sigma-Aldrich), fluconazole (Sigma-Aldrich), and amphotericin B (Sigma-Aldrich) were prepared in dimethyl sulfoxide (DMSO, Sigma-Aldrich). Caspofungin (Sigma-Aldrich) was dissolved in sterile water. The solutions were stored at −20 °C until use. The solvent concentrations in all tests did not exceed 5%.

### 3.2. Determination of Minimal Inhibitory Concentrations (MICs) 

The MICs of silymarin, caspofungin, fluconazole, and amphotericin B were determined with the broth microdilution method as recommended by CLSI, with some modifications [33]. A volume of 100 μL of RPMI-1640 medium was added to each well of a 96-well microplate and 100 μL of the test product was used to do a twofold serial dilution, giving concentrations of 3.9–2000 to µg/mL for silymarin, 0.02–10 µg/mL for caspofungin, and 0.012–100 µg/mL for fluconazole and amphotericin B. Stock inoculums were prepared by suspending planktonic cells grown to the exponential phase in Sabouraud liquid medium broth in sterile 0.85% NaCl and adjusting the turbidity to 1–5 × 10^6^ cells/mL at a 530 nm wavelength. The working suspension was prepared by making 1/200 dilution with RPMI of the stock suspension. A total of 20 μL of the inoculums were added to all wells, except for the negative control or blank. The negative control contained 100 μL of RPMI and 100 μL of the tested agent. In turn, the positive control contained the yeast suspension and RPMI medium. The plates were incubated for 48 h at 37 °C. MIC was visually determined and defined as the lowest concentration at which no growth was observed. Each experiment was performed in triplicate.

### 3.3. Effect of Silymarin on Candida Biofilm Formation and Preformed Biofilms

The reference strains of *Candida* were grown as biofilms using polystyrene flat-bottomed microtiter plates. The cell suspension was prepared in the RPMI-1640 medium at a cell density of 2 × 10^6^ cells/mL and dispensed into the wells of the microtiter plates (100 µL per well). The effect of silymarin on the biofilm formation ability was tested in the presence of 100 µL of different concentrations (MIC/16, MIC/8, MIC/4, and MIC/2). A total of 100 µL of the RPMI-1640 medium containing 5% DMSO without silymarin was the control. The plates were incubated for 48 h at 37 °C. At the end of incubation, the medium was aspirated from the wells and planktonic-phase cells were removed by washing the biofilms three times with PBS (phosphate-buffered saline, pH 7.4). The biofilms were dried at 60 °C for 2 h, and their biomasses were determined with the crystal violet assay described by Feoktistova M. et al. (2016) [34].

For preformed biofilms, the cell suspension was prepared in RPMI-1640 at a cell density of 1 × 10^6^ cells/mL, 100 µL of the cell suspension was dispensed into the wells, and incubated at 37 °C for two days. After incubation, non-adherent cells were gently removed and the wells were washed three times with PBS and filled with 100 µL of two-fold dilutions of silymarin in RPMI-1640 corresponding to MIC, 2× MIC, and 4× MIC. For the control, 100 µL of RPMI-1640 medium containing a final 5% DMSO were added into selected wells with biofilms. Furthermore, the microtiter plates were incubated at 37 °C for two days. After incubation in these conditions, the medium was aspirated from the wells and nonadherent cells were removed by washing the biofilms as described previously. The crystal violet staining of dry biofilms was performed according to method cited above. Each experiment was repeated three times as independent assays. 

### 3.4. Effect on Phospholipase Activity

To determine the effect of sylimarin on phospholipase activity, the *C. albicans* cells were incubated in the presence of sylimarin at concentrations corresponding to MIC/8, MIC/4, and MIC/2. After 20-h incubation at 37 °C on a shaker, the cells were harvested and the agent was removed by two cycles of dilution with sterile PBS and centrifugation for 10 min at 3000× *g*. The yeast pellets were resuspended in sterile PBS. The final suspension was adjusted to 1 × 10^8^ cells/mL.

The inhibition of phospholipase secretion was detected using the egg yolk agar plate method [35]. The medium was slightly modified by enriching Sabouraud dextrose agar (13 g) with NaCl (11.7 g), CaCl_2_ (0.11 g), and sterile egg yolk (10%), all in a final volume of 184 mL distilled water. *C. glabrata* was used for the negative control. Standard inoculums of the tested *Candida* (10 µL, with 10^8^ yeast cells/mL PBS) were deposited onto the egg yolk agar medium and left to dry at room temperature. The inoculated plates were then incubated at 37 °C for 48 h. The precipitation zone around the colony was observed for the production of the phospholipase enzyme. The phospholipase index was designed as P_z_ = *a/b*, where *a* is the diameter of the colony plus the precipitation zone and *b* is the diameter of the colony. Low P_z_ values indicate high enzymatic production and high P_z_ values represent low enzymatic production [36]. All experiments were performed in triplicate.

### 3.5. Inhibition of Proteinase Secretion

The influence of silymarin on the secretion of proteases by *C. albicans* was determined with the casein assay, which was conducted according to the method described by Ramesh et al. (2011) [37]. *Candida albicans* cells were grown under silymarin pressure at the same concentrations and conditions as described above. A cell free supernatant (1 mL) was added to 1 mL of casein in phosphate buffer (pH 7.5) and incubated for 1 h. The reaction was stopped by adding 0.2 M TCA (trichloroacetic acid); then, the reaction mixture was centrifuged at 3000 rpm for 10 min. The supernatant was added to 0.55 M sodium carbonate and Folin–Ciocalteu reagent. The mixture was incubated for 15 min. Next, optical density (OD) values were measured at 650 nm against the control. 

The content of proteinases in the control culture (without silymarin) was defined as 100%. Loss of enzyme activity induced by silymarin was presented as a percentage in relation to the control. All experiments were repeated three times.

### 3.6. Relative Quantification by Real-Time Reverse Transcriptase (RT)- PCR

The expression of the SAP4 gene was evaluated using real-time PCR after treatment with silymarin. The cells of *C. albicans* ATCC 10231 were exposed to silymarin at the concentration of 15 µg/mL (MIC/2) or 1% DMSO (control) during propagation in liquid culture in Spider medium (1% nutrient broth, 1% mannitol, 0.2% K_2_PO_4_ (Sigma-Aldrich), pH 7.2) with 10% fetal bovine serum (FBS) (Sigma-Aldrich). After 20-h incubation at 37 °C, the yeasts were collected and total RNA was extracted with the YeaStar RNA kit (Zymo Research, USA), according to the manufacturer’s instructions. cDNA was then synthesized using a Smart First Strand cDNA Synthesis Kit (EurX, Poland) following the manufacturer’s instructions. For PCR detection of transcripts, we used TaqMan gene expression assays (Lot: 170255, designed by manufacturer, Thermo Fisher Scientific, England) and the Probe qPCR Master Mix (EurX, Poland). The cDNA samples were pre-treated at 50 °C for 2 min with uracil-N-glycosylase to degrade any dUMP-containing PCR products and then subjected to initial denaturation at 95 °C for 10 min., followed by 40 amplification cycles with denaturation at 94 °C for 15 s, annealing at 60 °C for 30 s, and extension at 72 °C for 30 s using RotorGene-6000 (Corbett). The relative level of expression of the tested gene was calculated with the 2^-(ΔΔCt)^ method using *ACT1* as a reference gene [38].

### 3.7. In Vitro Activity of Antifungal Combinations against Candida Species

The effectiveness of the combinations of silymarin and commercial antibiotics was determined using the Checkerboard microdilution method [39].

Amphotericin B/silymarin, caspofungin/silymarin, and fluconazole/silymarin were used at 1/32, 1/16, 1/8, 1/4 1/2, MIC, 2× MIC, and 4× MIC of each strain. The MICs of *Candida* spp. were determined using the CLSI broth microdilution method as described previously. Antifungal interactions were determined referring to the fractional inhibitory concentration index (FICI). The FICI values were calculated for each well with the equation FICI = FICA + FICB = (MICA + B/MICA) + (MICB + A/MICB), where MICA and MICB are the MICs of drugs A and B alone, respectively, and MICA + B and MICB + A are the concentrations of the drugs applied in combination, respectively, in all the wells corresponding to the MIC. A combination of two drugs is considered synergistic when the FICI is ≤0.5, indifferent when the FICI is >0.5 to ≤4, and antagonistic when the FICI is >4. All experiments were performed in triplicate [40].

## 4. Conclusions

This investigation provides more information on silymarin as an antifungal substance. In addition to the known activities of sylimarin, this compound can act as an anti-*Candida* agent targeting mature biofilm and the secretion of phospholipases and proteinases, which are very important virulence factors. Finally, we have demonstrated that combinations of silymarin with antifungal antibiotics amphotericin B, fluconazole, and caspofungin do not cause undesired antagonistic interactions against reference strains of *Candida*. These findings suggest that silymarin may be useful in the treatment of candidiasis in the future.

## Figures and Tables

**Table 1:** Minimal inhibitory concentrations (µg/mL) of silymarin against Candida spp.

Strain	Silymarin	Amphotericin B	Caspofungin	Fluconazole
*C. albicans*	30	3.12	0.09	0.39
*C. parapsilosis*	60	6.25	0.78	0.39
*C. glabrata*	300	6.25	0.19	No activity
*C. tropicalis*	300	12.5	0.09	3.12
*C. krusei*	30	12.5	0.39	No activity

**Table 2:** In vitro activity of silymarin in combination with amphotericin B, caspofungin, and fluconazole determined by the broth dilution assay against *Candida* spp.

Strain	Amphotericin B + Silymarin		Caspofungin + Silymarin		Fluconazole + Silymarin	
	FICI	Activity	FICI	Activity	FICI	Activity
*C. albicans*	1.5	I	0.74	I	2.0	I
*C. parapsilosis*	1.5	I	1.0	I	2.0	I
*C. glabrata*	1.5	I	2.0	I	Not studied	-
*C. tropicalis*	2.0	I	2.0	I	2.0	I
*C. krusei*	0.35	S	0.39	S	Not studied	-

FICI—Fractional Inhibitory Concentration Index, S—synergism, I—indifference.

## References

[1] Bunchorntavakul C., Reddy K.R. (2013). Review article: Herbal and dietary supplement hepatotoxicity. Aliment. Pharmacol. Ther..

[2] Abenavoli L., Izzo A.A., Milić N., Cicala C., Santini A., Capasso R. (2018). Milk thistle (*Silybum marianum*): A concise overview on its chemistry, pharmacological, and nutraceutical uses in liver diseases. Phytother. Res..

[3] Kvasnicka F., Bíba B., Sevcík R., Voldrich M., Krátká J. (2003). Analysis of the active components of silymarin. J. Chromatogr. A.

[4] Pradhan S.C., Girish C. (2006). Hepatoprotective herbal drug, silymarin from experimental pharmacology to clinical medicine. Indian J. Med. Res..

[5] Dadar M., Tiwari R., Karthik K., Chakraborty S., Shahali Y., Dhama K. (2018). Candida albicans–Biology, molecular characterization, pathogenicity, and advances in diagnosis and control–An update. Microb. Pathog..

[6] Sardi J.C., Scorzoni L., Bernardi T., Fusco-Almeida A.M., Mendes Giannini M.J. (2013). *Candida species*: Current epidemiology, pathogenicity, biofilm formation, natural antifungal products and new therapeutic options. J. Med. Microbiol..

[7] Zacchino S.A., Butassi E., Liberto M.D., Raimondi M., Postigo A., Sortino M. (2017). Plant phenolics and terpenoids as adjuvants of antibacterial and antifungal drugs. Phytomedicine.

[8] Wagoner J., Negash A., Kane O.J., Martinez L.E., Nahmias Y., Bourne N., Owen D.M., Grove J., Brimacombe C., McKeating J.A. (2010). Multiple Effects of Silymarin on the Hepatitis C Virus Lifecycle. Hepatology.

[9] McClure J., Margineantu D.H., Sweet I.R., Polyak S.J. (2014). Inhibition of HIV by Legalon-SIL is independent of its effect on cellular metabolism. Virology.

[10] Qi F.H., Wang Z.X., Cai P.P., Zhao L., Gao J.J., Kokudo N., Li A.Y., Han J.Q., Tang W. (2013). Traditional Chinese medicine and related active compounds: A review of their role on hepatitis B virus infection. Drug Discov. Ther..

[11] Rakelly de Oliveira D., Tintino R., Braga M.F.B.M., Boligon A.A., Athayde M.L., Coutinho H.D.M., de Menezes I.R.A., Fachinetto R. (2015). In Vitro Antimicrobial and Modulatory Activity of the Natural Products Silymarin and Silibinin. BioMed Res. Int..

[12] Lee J.S., Hong D.Y., Kim E.S., Lee H.G. (2017). Improving the water solubility and antimicrobial activity of silymarin by nanoencapsulation. Colloids Surf. B Biointerfaces.

[13] Yun D.G., Lee D.G. (2017). Silymarin exerts antifungal effects via membrane-targeted mode of action by increasing permeability and inducing oxidative stress. Biochim. Biophys. Acta Biomembr..

[14] Kareem S.M., Mahmood S.S., Hindi N.K. (2019). Effects of Curcumin and Silymarin on the Shigella dysenteriae and Campylobacter jejuni In vitro. J. Gastrointest. Cancer.

[15] Mayer F.L., Wilson D., Hube B. (2013). *Candida albicans* pathogenicity mechanisms. Virulence.

[16] Dutreix L., Bernard C., Juin C., Imbert C., Girardot M. (2018). Do raspberry extracts and fractions have antifungal or anti-adherent potential against *Candida* spp.?. Int. J. Antimicrob. Agents.

[17] Evensen N.A., Braun P.C. (2009). The effects of tea polyphenols on *Candida albicans*: Inhibition of biofilm formation and proteasome inactivation. Can. J. Microbiol..

[18] Janeczko M., Masłyk M., Kubiński K., Golczyk H. (2017). Emodin, a natural inhibitor of protein kinase CK2, suppresses growth, hyphal development, and biofilm formation of *Candida albicans*. Yeast.

[19] Wall G., Montelongo-Jauregui D., Vidal Bonifacio B., Lopez-Ribot J.L., Uppuluri P. (2019). *Candida albicans* biofilm growth and dispersal: Contributions to pathogenesis. Curr. Opin. Microbiol..

[20] Donlan R.M. (2002). Biofilms: Microbial life on surfaces. Emerg. Infect. Dis..

[21] Zacchino S.A., Butassi E., Cordisco E., Svetaz L.A. (2017). Hybrid combinations containing natural products and antimicrobial drugs that interfere with bacterial and fungal biofilms. Phytomedicine.

[22] Kadir T., Gümrü B., Uygun-Can B. (2007). Phospholipase activity of *Candida albicans* isolates from patients with denture stomatitis: The influence of chlorhexidine gluconate on phospholipase production. Arch. Oral. Biol..

[23] Mavor A.L., Thewes S., Hube B. (2005). Systemic fungal infections caused by *Candida species*: Epidemiology, infection process and virulence attributes. Curr. Drug Targets.

[24] Price M.F., Wilkinson I.D., Gentry L.O. (1982). Plate method for detection of phospholipase activity in *Candida albicans*. Sabouraudia.

[25] Naglik J.R., Challacombe S.J., Hube B. (2003). *Candida albicans* secreted aspartyl proteinases in virulence and pathogenesis. Microbiol. Mol. Biol. Rev..

[26] Hube B., Naglik J. (2001). *Candida albicans* proteinases: Resolving the mystery of a genefamily. Microbiology.

[27] Hube B., Monod M., Schofield D.A., Brown A.J., Gow N.A. (1994). Expression of seven members of the gene family encoding secretory aspartyl proteinases in *Candida albicans*. Mol. Microbiol..

[28] White T.C., Agabian N. (1995). *Candida albicans* secreted aspartyl proteinases: Isoenzyme pattern is determined by cell type, and levels are determined by environmental factors. J. Bacteriol..

[29] Chatrath A., Gangwar R., Kumari P., Prasad R. (2019). In Vitro Anti-Biofilm Activities of Citral and Thymol Against Candida Tropicalis. J. Fungi.

[30] Saibabu V., Singh S., Ansari M.A., Fatima Z., Hameed S. (2017). Insights into the intracellular mechanisms of citronellal in *Candida albicans*: Implications for reactive oxygen species-mediated necrosis, mitochondrial dysfunction, and DNA damage. Rev. Soc. Bras. Med. Trop..

[31] El Zawawy N.A., Hafez E.E. (2017). Efficacy of Pluchea dioscoridis leaf extract against pathogenic *Candida albicans*. J. Infect. Dev. Ctries.

[32] Cui J., Ren B., Tong Y., Dai H., Zhang L. (2015). Synergistic combinations of antifungals and anti-virulence agents to fight against *Candida albicans*. Virulence.

[33] CLSI (2008). Reference Method for Broth Dilution Antifungal Susceptibility Testing of Yeasts, Vol 28, No. 14. Approved Standard.

[34] Feoktistova M., Geserick P., Leverkus M. (2016). Crystal Violet Assay for Determining Viability of Cultured Cells. Cold Spring Harb. Protoc..

[35] Samaranayake L.P., Raeside J.M., MacFarlane T.W. (1984). Factors affecting the phospholipase activity of Candida species in vitro. Sabouraudia.

[36] Anil S., Samaranayake L.P. (2003). Brief exposure to antimycotics reduces the extracellular phospholipase activity of *Candida albicans* and *Candida tropicalis*. Chemotherapy.

[37] Ramesh N., Priyadharsini M., Sumathi C.S., Balasubramanian V., Hemapriya J., Kannan R. (2011). Virulence Factors and Anti-Fungal Sensitivity Pattern of Candida Sp. Isolated from HIV and TB Patients. Indian J. Microbiol..

[38] Cao Y., Dai B., Wang Y., Huang S., Xu Y., Cao Y., Gao P., Zhu Z., Jiang Y. (2008). In vitro activity of baicalein against *Candida albicans* biofilms. Int. J. Antimicrob. Agents.

[39] Odds F.C. (2003). Synergy, antagonism, and what the chequerboard puts between them. J. Antimicrob. Chemother..

[40] Petersen P.J., Labthavikul P., Jones C.H., Bradford P.A. (2006). In vitro antibacterial activities of tigecycline in combination with other antimicrobial agents determined by chequerboard and time-kill kinetic analysis. J. Antimicrob. Chemother..

